# 131. Carbapenemase-producing *Acinetobacter* spp. from Israel, 2001-2006: earliest report of *bla*_NDM_ predating the oldest known *bla*_NDM_-positive strains

**DOI:** 10.1093/ofid/ofac492.209

**Published:** 2022-12-15

**Authors:** Louis-Patrick Haraoui, Frédéric Grenier, Félix Heynemand, Simon Lévesque, Richard Sullivan, Hannah L Landecker, Paul G Higgins, Sébastien Rodrigue

**Affiliations:** Université de Sherbrooke, Quebec, Canada; Université de Sherbrooke, Quebec, Canada; Université de Sherbrooke, Quebec, Canada; Université de Sherbrooke, Quebec, Canada; King's College London, London, England, United Kingdom; UCLA, Los Angeles, California; University of Cologne, Cologne, Nordrhein-Westfalen, Germany; Université de Sherbrooke, Quebec, Canada

## Abstract

**Background:**

Carbapenem-resistant *Acinetobacter baumannii* (CRAb) is a WHO priority 1 critical pathogen. Despite Israel being affected early by high CRAb rates, limited molecular data are available. We investigated the presence of carbapenemases among 198 *Acinetobacter* spp. clinical isolates from Israel between 2001 and 2006.

**Methods:**

Strains from 3 archives underwent whole-genome sequencing (Illumina NovaSeq on all, MinION on a subset) and computational analyses: assembly (Unicycler), annotation (prokka), identification (Kraken, 16S rRNA), search for carbapenemases (ResFinder, BLDB curation). Figures were generated in Inkscape, plasmid alignment on AliTV.

**Results:**

*A. baumannii* (Ab) represented 179/198 (90.4%) *Acinetobacter* spp. (Figure 1). Annual incidence varied from a minimum of 16 (2001) to a maximum of 62 (2004), with an average of 30. Eighty-four Ab (46.9%) carried a carbapenemase: 38 (45.2%) *bla*_OXA-72_ (*bla*_OXA-24-like_); 28 (33.3%) *bla*_OXA-23-like_ (20 *bla*_OXA-23_ and 8 *bla*_OXA-225_); 18 (21.5%) *bla*_OXA-58_ (16 from 2001-2). Annual CRAb rate increased yearly from 2002 (32%) to 2006 (67%).

Eight species of non-*baumannii Acinetobacter* (NbA) accounted for 19 isolates (9.6%): *A. pittii* (n=6), *lwoffii* (n=4), *junii* (n=3), *ursingii* (n=2), others n=1: *A. gyllenbergii*, *johnsonnii*, *schindleri*, and *variabilis* (Figure 1). Two of three *A. junii* contained *bla*_OXA-58_, one of which, Ajun-H1-3, isolated in a blood culture in January 2004, also possessed *bla*_NDM-1_. The pNDM-Ajun-H1-3 plasmid matched numerous NDM-positive plasmids reported from 2005 onwards in *Acinetobacter* spp. as well as *Enterobacterales* (Figure 2).

**Figure 1:**
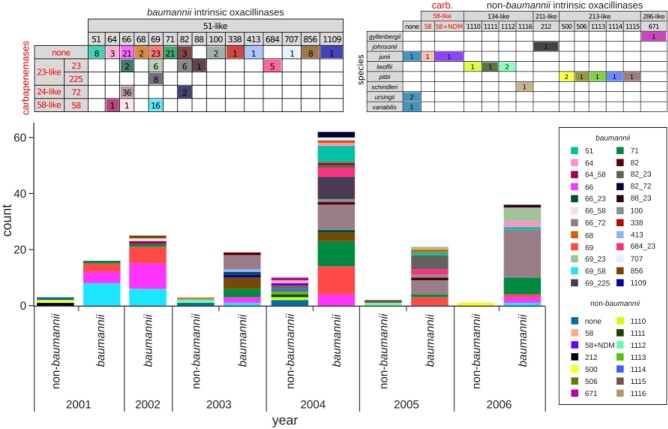
annual distribution of Acinetobacter baumannii and non-baumannii Acinetobacter in Israel between 2001 and 2006, including their intrinsic oxacillinases and the presence of carbapenemases.

**Figure 2:**
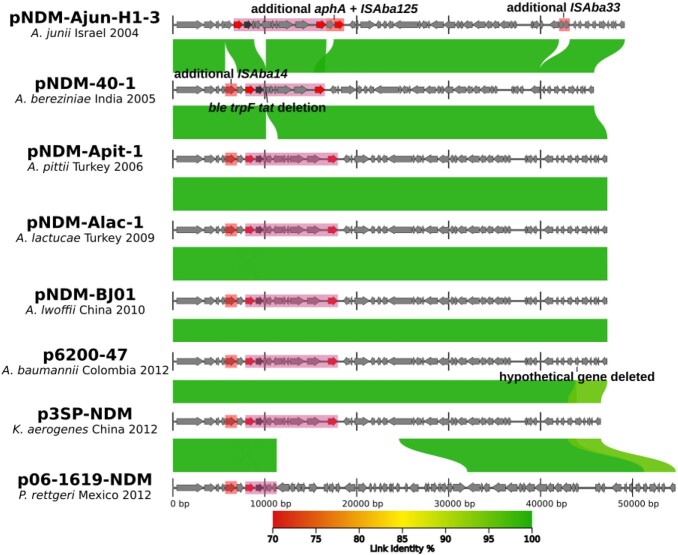
plasmid alignment of pNDM-Ajun-H1-3 (Israel, 2004) with other NDM-positive plasmids found globally from 2005 onwards in Acinetobacter spp. and Enterobacterales.

**Conclusion:**

We retrospectively assessed carbapenemase diversity among *Acinetobacter* spp. in Israel from 2001-2006. Analysis of 179 Ab isolates predate observations elsewhere: rapidly rising CRAb rates, driven by the dissemination of *bla*_OXA-23-like_ and *bla*_OXA-24-like_ genes replacing *bla*_OXA-58_.

Among 19 NbA, an *A. junii* isolated in 2004 carried two carbapenemases, *bla*_OXA-58_ and *bla*_NDM-1_, making it the earliest NDM-positive isolate reported to date, preceding NDM-positive *Acinetobacter* spp. found in 2005 in India.

Further investigations into the origins of *bla*_NDM_ are needed to understand the conditions that led to its emergence and prevent similar issues from arising in the future.

**Disclosures:**

**Paul G. Higgins, PhD**, Coris Bioconcept: Supply antibodies for lateral flow test kits.

